# Myocarditis in a Pediatric Patient with *Campylobacter* Enteritis: A Case Report and Literature Review

**DOI:** 10.3390/tropicalmed6040212

**Published:** 2021-12-16

**Authors:** Anastasios-Panagiotis Chantzaras, Spyridon Karageorgos, Panagiota Panagiotou, Elissavet Georgiadou, Theodora Chousou, Kalliopi Spyridopoulou, Georgios Paradeisis, Christina Kanaka-Gantenbein, Evanthia Botsa

**Affiliations:** 1 First Department of Pediatrics, Medical School, National and Kapodistrian University of Athens, “Aghia Sophia” Children’s Hospital, Thivon and Papadiamantopoulou Street, 11527 Athens, Greece; tasosxantz@med.uoa.gr (A.-P.C.); s.karageorgos@external.euc.ac.cy (S.K.); panagpan@med.uoa.gr (P.P.); apanepistimiaki@paidon-agiasofia.gr (E.G.); dunivped@paidon-agiasofia.gr (T.C.); ckanaka@med.uoa.gr (C.K.-G.); 2 Department of Microbiology, National and Kapodistrian University of Athens, “Aghia Sophia” Children’s Hospital, Thivon and Papadiamantopoulou Street, 11527 Athens, Greece; micro@paidon-agiasofia.gr (K.S.); Proora5@paidon-agiasofia.gr (G.P.)

**Keywords:** myocarditis, myopericarditis, *Campylobacter*, pediatric, review

## Abstract

Myocarditis represents a potential complication of various infectious and noninfectious agents and a common diagnostic challenge for clinicians. Data regarding *Campylobacter*-associated myocarditis are limited. Here, a case of a 13-year-old female with *Campylobacter* jejuni gastroenteritis complicated by myocarditis is presented, followed by a literature review in order to retrieve information about *Campylobacter*-associated carditis in the pediatric population. A search on MEDLINE/PubMed yielded 7relevant cases in the last 20 years. Most of them (six/seven) were males and the mean age was 16.1 years. All patients presented with gastrointestinal symptoms followed in six/seven cases by chest pain within two to seven days. *Campylobacter* was isolated from stool cultures in six patients; abnormal electrocardiographic findings were detected in six; and abnormal echocardiographic findings in three of the cases. Five patients were treated with antibiotics. Full recovery was the clinical outcome in six patients, whereas one patient died. Concerning the nonspecific symptoms of patients with myocarditis, high clinical suspicion of this complication is necessary in cases where patients with a recent infection present with chest pain and elevated cardiac biomarkers.

## 1. Introduction

Myocarditis is defined as the inflammation of the heart muscle and represents a potential complication of various infectious and noninfectious agents. Recently, the development of sensitive diagnostic tests, such as polymerase chain reaction (PCR) assays, led to the isolation of responsible pathogens; viral causes continue to be the most prevalent among all causes [1]. Bacterial infections are a rare cause of myocarditis, with gastrointestinal pathogens including *Salmonella* spp. and *Shigella* spp. being commonly reported. A few cases of myocarditis as a complication of *Campylobacter jejuni* infection have been reported in adults thus far [2].

Here, we present a case of a healthy female adolescent who was diagnosed with *C. jejuni*-associated myocarditis. We also present the results of a literature review regarding *Campylobacter*-associated carditis in the pediatric population (<18 years old).

## 2. Case Report

A 13-year-old previously healthy and fully vaccinated female was admitted to the Emergency Department (ED) of “Aghia Sophia” Children’s Hospital of Athens in July. She complained of fever (Tmax = 38.5 °C), intermittent diffuse abdominal pain, and non-bloody diarrhea for the last two days. She also reported headaches and nuchal ache during active movement. A few days before the onset of diarrhea, she had eaten poultry in a local restaurant. There were no other family members with gastrointestinal symptoms. On examination, the patient was in good general condition, afebrile (T = 37.2 °C), with BP = 118/62 mmHg, HR = 123 bpm, RR = 18/min, and SpO_2_ = 98% on room air. There were no signs of nuchal rigidity, photophobia, and pupils were equal and reactive. Lungs were clear to auscultation, cardiac auscultation revealed normal S_1_, S_2_ with no murmurs, gallops, or pericardial friction rub, and distal pulses were symmetrical and equal. Abdominal examination revealed diffuse tenderness upon deep palpation with no signs of guarding. The review of the other systems was grossly normal. The patient was admitted to the General Pediatric Ward with the working differential diagnosis of infectious enteritis and acute appendicitis; patient’s neurological examination was normal, Kernig’s and Brudzinski’s signs were negative, and therefore meningitis was low in our differential diagnosis list. Surgical consultation was requested, which did not show any signs of acute abdomen, no evident signs of appendicitis, with McBurney’s sign being negative.

Initial laboratory investigations revealed normal complete blood count, electrolytes, renal, and liver function tests, whereas serum C-reactive protein value was elevated at 25 mg/dL (reference <0.5 mg/dL). Urinalysis was normal whereas blood, urine, and stool cultures were sent.

**Figure 1:**
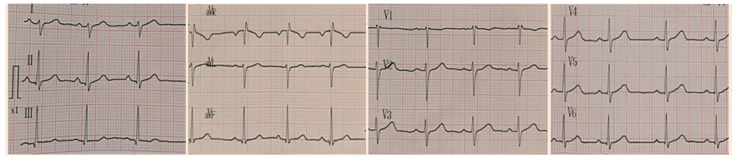
ECG on Day 2.

**Figure 2:**
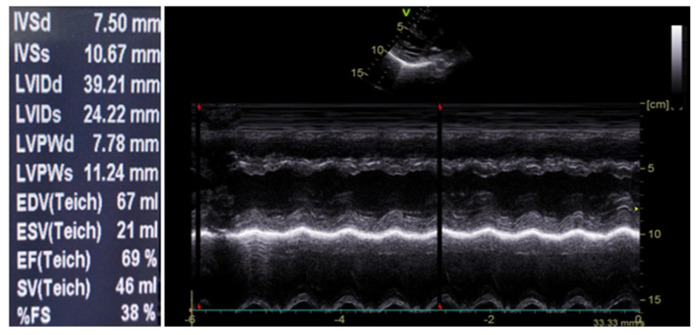
Cardiac echocardiography on Day 2.

Upon admission, the patient received intravenous maintenance fluids and remained in good general condition. During the second day of hospitalization, she had three episodes of fever (Tmax = 38.2 °C) and started to complain about stabbing, intermittent pain at the anterior chest, without radiation. There were no alleviating or aggravating factors. Her physical examination was unremarkable. Laboratory investigation including cardiac biomarkers were sent and showed elevated total Creatine Kinase (CK) (307 IU/L; reference range <140), CK-MB (25.3 ng/mL; reference <3), and Τroponin-T (456.4 pg/mL; reference< 14) levels (Table 1). An electrocardiogram (ECG) was performed that showed sinus rhythm, normal ST-segments, and normal T waves (Figure 1). Due to persisting chest pain and due to the abnormal cardiac biomarkers, a cardiological consultation was requested. Cardiac echocardiography was performed that showed normal left ventricular (LV) function, with a >65% ejection fraction and no pericardial effusion (Figure 2).

An extensive laboratory and microbiological evaluation on a working diagnosis of acute myocarditis were performed. A rapid multiplex PCR stool analysis using the BioFire^®^ FilmArray^®^ Gastrointestinal (GI) panel was sent identifying *Campylobacter* spp. Subsequently, further laboratory evaluation for common cardiotropic infectious causes was negative (Table 2). Therefore, with the diagnosis of suspected/potential acute myocarditis associated with *Campylobacter* spp. enterocolitis the patient was treated with a five-day antibiotic course of azithromycin (initial dose of 10 mg/kg followed by 5 mg/kg once daily for 4 days), and antiarrhythmic medication of carvedilol (0.05 mg/kg BD) and captopril (6.25 mg BD). Immunomodulatory or immunosuppressive therapies, including corticosteroids, intravenous immunoglobulin (IVIG), azathioprine, and cyclosporine reported in the literature [1] were not part of our therapeutic approach for our patient. Later on during hospitalization, fecal cultures were negative for common bacterial and viral agents, whereas *Campylobacter jejuni* was isolated which was resistant to fluoroquinolones and sensitive to macrolides and tetracyclines. Blood cultures were negative.

During hospitalization, the patient remained in good general condition and was hemodynamically stable. In order to closely monitor her cardiovascular status, repeated electrocardiographic and echocardiographic evaluations, as well as a continuous 24 h cardiac rhythm Holter monitoring, were performed (all of which were normal). She remained afebrile and diarrhea stopped during the third day of hospitalization. Chest pain resolved by the fourth in-hospital day. Regarding her laboratory tests, CK value peaked at 643 IU/L, CK-MB to 51.6 ng/mL, Troponin-T to 872.2 ng/mL, N-terminal-proBNP to 715 pg/mL, AST to 64 IU/L and C-reactive protein to 48 mg/L. The patient was discharged in a good clinical condition after eight days of hospitalization with a follow-up appointment in order to have cardiac magnetic resonance imaging (CMR) and cardiologic evaluation.

**Figure 3:**
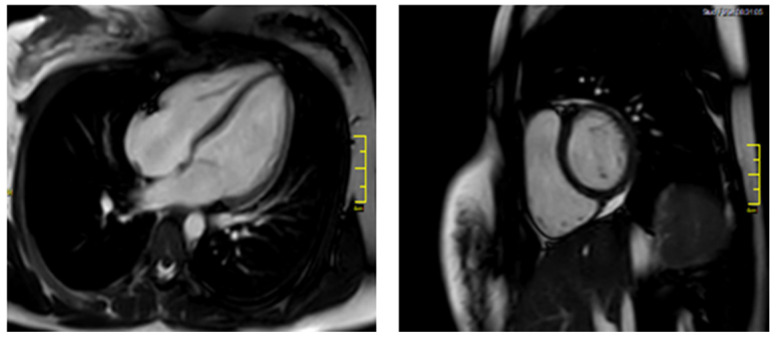
Follow-up CMR.

On the follow-up appointment, one month after discharge, patient’s electrocardiographic and echocardiographic evaluations as well as serum cardiac biomarkers were normal. Additionally, the CMR findings did not demonstrate any markers of myocardial inflammation and necrosis (Figure 3). The Luise Lake Criteria for acute myocarditis were not fulfilled by that time as no high signal intensity on T2-weighted imaging, increased T2 and T1 times, myocardial thickening, or rapid uptake (early gadolinium enhancement) of contrast were revealed [3]. Subsequently, antiarrythmic medication was discontinued.

## 3. Discussion and Literature Review

In this case report, we present a previously healthy young female patient, who was admitted with gastroenteritis symptoms and within 24 h from admission developed chest pain. Based on the clinical symptoms, elevated cardiac biomarkers, the isolation of *Campylobacter jejuni* from both stool culture and PCR array, as well as the lack of potential alternative diagnosis, she was diagnosed with suspected/potential *Campylobacter jejuni*–associated myocarditis in accordance with AHA statement [1]. Our patient represents the third case of *C. jejuni*-associated myocarditis and the second female case reported in the literature. Importantly, both electrocardiographic and echocardiographic findings of our patient were normal, the patient remained in good clinical condition and full recovery was the clinical outcome. The cardiological follow-up evaluation one month later was also normal.

A search on MEDLINE/PubMed using multiple combinations of MESH-terms “*Campylobacter*”, “*Campylobacter jejuni*”. “myocarditis”, “myopericarditis”, and “pericarditis” yielded seven relevant cases during the last 20 years in the pediatric population (<18 years old). Specifically, four cases of myopericarditis [4,5,6], two cases of myocarditis [7,8], and one case of pericarditis [9] associated with *Campylobacter* spp. infection have been described. Patients’ clinical characteristics are provided in Table 3.

Regarding the characteristics of the patients included in this review, most of them were males (six/seven patients) and the mean age was 16.1 years. Past medical history of the patients was as follows; one patient had β-thalassemia [9], one patient had a previous episode of myopericarditis three years before the current episode [4], one patient suffered from bronchial asthma [8], whereas no comorbidities were reported for the rest of the patients.

All patients presented with early onset gastrointestinal tract symptoms followed by clinical symptoms of carditis within two to seven days. Chest pain was the main complaint of carditis in the patients described, whereas only one [9] did not have any cardiac symptoms. Laboratory tests demonstrated the release of cardio-specific enzymes (CK, CK-MB, Troponin T/I) in all patients (*n* = 6) tested for cardiac biomarkers [4,5,6,7,8].

Electrocardiographic and echocardiographic evaluation were performed in six out of seven patients as long as cardiac symptoms were present [4,5,6,8,9]. Abnormal ECG findings were detected in 6 [4,5,6,8,9]. Specifically, isolated ST-segment elevations [4,5,8,9] or combined with T waves inversions [6] were commonly reported. Abnormal echocardiographic findings were present in three patients, including impaired left ventricular function (reduced ejection fraction) in two of them [4,5], and large pericardial effusion in the third one [9]. One patient [6] had normal transthoracic echocardiography. In two cases [5,6] myocardial damage was detected in a CMR conducted early after the onset of cardiac symptoms, confirming the diagnosis. Despite the fact that endomyocardial biopsy (EMB) is the gold standard to prove the diagnosis of myocarditis [1], it was used only in one case report [7] as part of the autopsy. Low sensitivity due to sampling error, variability in pathologic interpretation, and potential complications such as myocardial perforation and tamponade are reasons for its reduced use [3].

The diagnosis of *Campylobacter* infection was based on the isolation of the microbe from stool cultures in all patients, except for one where *Campylobacter* was isolated from the pericardial fluid [9]. In six of the cases stool isolate was *Campylobacter jejuni*, whereas *Campylobacter fetus* was detected in one case [9].

Antibiotics were part of the therapeutic approach in five out of the seven patients in this review [4,5,6,9]. Concerning clinical outcomes, six patients fully recovered [4,5,6,8,9], whereas one death was recorded [7].

Acute myocarditis represents a potentially life-threatening diagnosis and a common diagnostic challenge for clinicians due to the non-specific nature of presenting symptoms such as chest pain and fever. Recently, the American Heart Association published a scientific statement concerning the diagnosis, etiological factors, and management of myocarditis in children. Among commonly isolated pathogens, viral infections appear the most prevalent, including Enteroviruses (Coxsackie A and B, Echoviruses), Epstein–Barr virus (EBV), Cytomegalovirus (CMV), Human Herpes Virus 6 (HHV6), Adenoviruses, Influenza A and B, Parvovirus B19, and Hepatitis B and C. Additionally, bacterial and fungal agents, as well as immunοlogical and pharmaceutical agents are also reported as less common factors to cause myocarditis [1]. Of note, in this statement *Campylobacter* is not described as a potential etiological factor.

*Campylobacter* infections represent a common cause of gastroenteritis in children worldwide, and *Campylobacter jejuni* is the most commonly isolated *Campylobacter* spp. Typically, symptoms are relatively mild and *Campylobacter* gastroenteritis is usually self-limited. However, multiple acute and late complications including cholecystitis, reactive arthritis, Guillain-Barre syndrome (GBS), and rarely myocarditis and pericarditis have been reported in the literature [10,11,12,13,14]. The exact pathogenetic mechanism responsible for cardiac inflammation remains uncertain; potential mechanisms include—but are not limited to—direct bacterial invasion of cardiac tissue, the release of bacterial toxins, circulating immune complexes, and cytotoxic T-cells [15].

The results of this literature review highlight that *Campylobacter*-related cardiac complications predominately affect male adolescents, which is in line with the current reported literature regarding acute myo(peri)carditis in the pediatric population [16]. Importantly, most of the cases responded well to antibiotic and supportive treatment, following a benign course. Regarding initial clinical presentation, all patients presented with early gastrointestinal symptoms, followed by clinical symptoms of carditis within the next few days. Cardio-specific biomarkers were typically elevated, while electrocardiographic and echocardiographic changes were present in the majority of cases. These findings are more specific and contribute in clinical practice to the diagnosis. Of note, in two casesCMR was performed confirming the diagnosis of acute myocarditis [5,6].

In summary, this case highlights a patient with *Campylobacter jejuni* gastroenteritis that was subsequently complicated by clinical and laboratory manifestations of myocarditis. Therefore, high clinical suspicion of myocarditis is necessary in patients with a recent infection that present with chest pain and elevated cardiac biomarkers.

## Figures and Tables

**Table 1:** Laboratory parameters and cardiological tests.

		Reference Values	Emergency Department	Day 2	Day 3	Day 6	Day 8
Complete Blood Count	WBC (/μL)		11,220	9750	9150	9830	12,790
	Neu/Lymph/Mono (%)		80.6/10.8/7	78.6/12.5/8	74.6/13.5/8	51.6/32/9	67.2/22.6/6
	Hgb (mg/dL)/Hct (%)		12/37.2	11.8/35.9	11.3/33.9	12.3/39	12.2/38
	PLT (/μL)		226,000	205,000	185,000	306,000	298,000
Blood Metabolic Panel	U/Cr (mg/dL)	9–35/0.2–1	24/0.84	18/0.75	15/0.72	30/0.7	24/0.7
	AST/ALT (IU/L)	10–60/5–45	13/12	31/17	64/23	23/34	15/25
	LDH (IU/L)	120–300	168		285	268	190
	CK (IU/L)	<140		307	643	35	35
Cardiac Biomarkers	CK-MB (ng/mL)	<3		25.3	51.6	0.7	0.9
	Troponin T (pg/mL)	<14		456.4	872.2	24.4	8.2
	NT-proBNP (pg/mL)	<300			715	369	185
Inflammatory markers	ESR 1sth (mm)				55	45	
	CRP (mg/L)	<10	25.2	40.1	48.4	8.2	3.0
Cardiology	Electrocardiography			Normal	Normal	Normal	Normal
	Echocardiography			Normal	Normal	Normal	Normal
	24 h Holter monitoring					Normal	

NT-proBNP: N-terminal prohormone of brain natriuretic peptide; CK: creatine kinase; ESR: estimated sedimentation rate; CRP: C-reactive protein.

**Table 2:** Microbiological tests.

Test	Day 1	Day 2					
Urinalysis	PH = 6.5 SG = 1016 RBC = 0–2/cfu WBC = 0–2/cfu						
Stool culture	*Campylobacter jejuni*						
Blood culture	Negative						
Rapid multiplex PCR stool analysis	*Campylobacter* spp.						
Serological testing		EBV	IgM IgG	−	Parvovirus	IgM IgG	− −
				+			
		CMV	IgM IgG	− −	SARS-CoV-2	IgG	−
		HSV	IgM IgG	− −	Mycoplasma	IgM IgG	−
							+
		HHV-6	IgM IgG	− −	HBV	HBsAg	−
		HIV	Ag Ab	− −	HCV	Anti-HCV	−
Nasopharyngeal samples		Ag	Influenza A &B				−
			Adenovirus				−
		PCR	Coxsackie A				−
			Coxsackie B				−

SG, specific gravity; RBC, red blood cells; WBC, white blood cells; EBV, Epstein–Barr virus; CMV, Cytomegalovirus; HHV-6, Human Herpes Virus 6; HBV, Hepatitis B; HCV, Hepatitis C; HIV, Human Immunodeficiency Virus; Ag, antigen; Ab, antibody; PCR, polymerase chain reaction.

**Table 3:** Summary of the cases reported in the literature.

Author Year Country	G/ACardiac Involvement	GIS	CS (GIS-CS Interval)	ECG Changes	Echocardiographic Changes	Elevation of Cardiac Enzymes	Fecal Culture	Treatment (Outcome)
Yaita [8] 2020 Japan	M/16Myocarditis	Non hemorrhagic watery diarrhea, headache, fever, abdominal pain	Persistent chest pain at rest exacerbated by deep inspiration (4 days)	ST elevation in leads II, aVf, V3, V4, V5, and V6	No asynergy of ventricular movement or pericardial effusion	Troponin TCKNT-proBNP	*C. jejuni*	No antibiotics (Recovery)
Dind [4] 2019 Australia	M/17Myopericarditis	Diarrhea	Severe central chest pain (7 days)	Widespread concave-up ST elevation	Globally impaired left ventricle LVEF 40%	Troponin I	*C. jejuni*	AzithromycinColchicineIbuprofenParacetamol (Recovery)
Fica [6] 2010 Chile	M/17Myopericarditis	Upper abdominal pain, fever up to 38.5 °C, dysentery	Severe anteriorchest pain relieved by sitting (2 days)	ST-segment elevation on V1-V6 leads, and a negative T wave	Normal	Troponin ICKCK-MB	*C. jejuni*	Azithromycin (Recovery)
Heinzl [5] 2009 Austria	M/16Myopericarditis	Bloody diarrhea, fever up to 40 °C, headache, abdominal pain	Constant chest pain associated with dyspnea (5 days)	Sinus rhythm and significant ST elevation in leads I, II, V4, V5, and V6.	Reduced leftLVEF 45%,shortening fraction 22%	Troponin TCKCK-MB	*C. jejuni*	Clarithromycin (Recovery)
	M/17Myopericarditis	Fever up to 38 °C, diarrhea	Recurrent chest pain radiating to left arm (2 days)	Sinus rhythm and significant ST elevation in leads I, AVL, V4, and V5	Normal left ventricular function without localized areas of hypokinesis, no significant pericardial effusion	Troponin TCKCK-MB	*C. jejuni*	ClarithromycinMefenamic acid (Recovery)
Pena [7] 2007 USA	M/16Myocarditis	Fever, chills, myalgias, abdominal cramps, body stiffness, diarrhea, vomiting, decreasedappetite, sweats, abdominal tightness, restlessness	Chest tightness on inspiration, difficulty in breathing (4 days)	NR	NR	CK	*C. jejuni*	No antibiotics (Death)
Kanj [9] 2001 Lebanon	F/14Pericarditis	Fever, chills, vomiting, diarrhea	Severe dyspnea, dry cough (4 days)	Diffuse ST segment elevation	Large pericardial effusion, mild mitral regurgitation with significant respiratory variation across the mitral and the tricuspid valves, indicating tamponade	NR	*C. fetus*	Ampicillin (Recovery)
Present case 2021 Grreece	F/13Myocarditis	Fever, headache, abdominal pain, diarrhea	Stabbing pain of the anterior chest	Normal	Normal	Troponin TCKCK-MBNT-proBNP	*C. jejuni*	Azithromycin Carvedilol Captopril

G, gender; A, age in years; M, male; F, female; GIS, gastrointestinal symptoms; CS, cardiac symptoms; LVEF, left ventricular ejection fraction; CK, creatine kinase: NR, not reported; NT-proBNP, N-terminal prohormone of brain natriuretic peptide.
